# Concurrent Detection of Streptococcus pneumoniae and Herpes Simplex Virus Type 1 (HSV-1) in Cerebrospinal Fluid: Clinical Interpretation Beyond Molecular Positivity

**DOI:** 10.7759/cureus.103919

**Published:** 2026-02-19

**Authors:** Abdulmohsen Aljishi, Jumana M AlJishi, Fatimah M Alaqwal, Amal Alabbadi, Narjes M AlMousa

**Affiliations:** 1 Critical Care Medicine, Qatif Central Hospital, Qatif, SAU; 2 Internal Medicine, Qatif Central Hospital, Qatif, SAU; 3 Emergency Medicine, Imam Abdulrahman Bin Faisal University, Dammam, SAU

**Keywords:** central nervous system infection, encephalitis, herpes simplex virus type 1, pneumococcal meningitis, viral reactivation

## Abstract

Herpes simplex virus type 1 (HSV-1) encephalitis and acute bacterial meningitis are severe central nervous system infections associated with significant morbidity. While typically distinct, the increasing use of multiplex polymerase chain reaction (PCR) panels has led to occasional co-detection, creating diagnostic uncertainty. We describe a 44-year-old previously healthy male diagnosed with culture-confirmed pneumococcal meningitis in whom multiplex cerebrospinal fluid PCR also detected HSV-1. The clinical course was complicated by a generalized seizure on hospital day 3 and the development of ventriculitis on day 14, despite negative repeat microbiological assays. Following targeted antibacterial and antiviral therapy, the patient achieved complete neurological recovery. This case highlights the complexity of interpreting molecular results in the setting of severe blood-brain barrier disruption and emphasizes a cautious, chronologically driven approach to management.

## Introduction

Herpes simplex virus type 1 (HSV-1) encephalitis is the most common cause of sporadic viral encephalitis in adults globally, with an estimated annual incidence of two to four cases per million population [1]. Despite the availability of timely antiviral therapy, it remains associated with considerable morbidity and persistent neurocognitive or psychiatric sequelae [1,2]. The pathogenesis typically involves the reactivation of latent virus within the trigeminal or olfactory ganglia, often precipitated by stressors such as systemic inflammation or immune dysregulation [3].

Acute bacterial meningitis is similarly a life-threatening central nervous system (CNS) infection associated with significant mortality and long-term neurological impairment. *Streptococcus pneumoniae* remains the leading causative pathogen in community-acquired bacterial meningitis in adults, accounting for approximately 70%-75%. [4]. Diagnosis traditionally relies on cerebrospinal fluid (CSF) analysis, where the advent of multiplex polymerase chain reaction (PCR) platforms has revolutionized the rapid identification of bacterial and viral pathogens [5].

While HSV-1 and *S. pneumoniae* are generally regarded as distinct clinical entities, recent literature has begun to describe rare instances of viral detection during bacterial infection, which significantly complicates the therapeutic window [5]. In many reported cases, the clinical course follows a biphasic pattern: an initial response to antibacterial therapy followed by a delayed neurological decline consistent with viral encephalitis [5-7]. This suggests that the intense inflammatory milieu of bacterial meningitis may facilitate viral reactivation rather than mere coincidental infection [8,9].

However, the clinical significance of HSV-1 positivity in this context remains a subject of debate, specifically whether it represents true parenchymal encephalitis, secondary reactivation, or incidental molecular detection secondary to blood-brain barrier disruption.

## Case presentation

A 44-year-old previously healthy male presented to the emergency department with a two-day history of fatigue, low-grade fever, and cough, progressing to acute confusion. On examination, he was tachypneic and disoriented but hemodynamically stable without focal neurological deficits.

Initial laboratory investigations revealed significant leukocytosis and elevated inflammatory markers (Table 1). A non-contrast head CT was unremarkable. Empiric therapy was initiated with ceftriaxone, vancomycin, acyclovir, and dexamethasone.

**Table 1:** Laboratory values Reference ranges correspond to standard adult laboratory values. CRP, C-reactive protein; ESR, erythrocyte sedimentation rate; WBC, white blood cell.

Parameter	Unit	Reference range	Day 1	Day 3	Day 7	Day 14
Hemoglobin	g/dL	13.0-17.5	17.0	16.2	15.9	14.6
Platelets	×10^9^/µL	150-450	66	79	230	356
WBC	×10³/µL	4.0-11.0	19.2	19.4	13.4	8.2
Neutrophils	×10³/µL	1.5-7.5	15.7	13.9	10.7	7.2
Lymphocytes	×10³/µL	1.0-3.0	0.58	1.12	1.4	2.1
Urea	mmol/L	2.5-7.8	8.1	9.5	6.0	4.5
Creatinine	µmol/L	64-104	91	75	67	52
Sodium	mmol/L	135-145	138	141	130	133
Potassium	mmol/L	3.5-5.1	3.8	3.9	4.8	4.3
CRP	mg/L	<5	34.5	7.8	8.1	1.9
ESR	mm/h	<20	10	33	37	41

Lumbar puncture (LP) performed on day showed neutrophilic pleocytosis, markedly elevated protein (9.2 g/L), and severely low glucose (<0.2 mmol/L), consistent with bacterial meningitis (Table 2). Both CSF and blood cultures subsequently grew *S. pneumoniae*. Given the clear bacterial profile and early clinical stabilization, acyclovir was initially discontinued.

**Table 2:** Cerebrospinal fluid analysis Reference ranges correspond to standard adult laboratory values. RBC, red blood cell; WBC, white blood cell.

Parameter	Unit	Reference range	Day 1	Day 14
Polymorphs	%	0	100	80
Monomorphs	%	0	0	20
WBC	/mm³	0	130	185
RBC	/mm³	0	1500	50
Protein	g/L	0.2-0.4	9.2	Not performed
Glucose	mmol/L	2.2-3.9	<0.2	Not performed

On hospital day 3, the initial multiplex PCR (BioFire® FilmArray®) results returned positive for both *S. pneumoniae* and HSV-1. Concurrently, the patient suffered a generalized tonic-clonic seizure. Due to the temporal association between the seizure and the PCR results, intravenous acyclovir was reinitiated.

Subsequent brain MRI revealed multifocal T2/FLAIR hyperintensities with restricted diffusion in the bilateral thalami, brainstem, and medial temporal regions. These were interpreted as inflammatory changes secondary to severe CNS infection.

On day 14, despite clinical improvement, the patient developed recurrent fever and a decline in consciousness (Glasgow Coma Scale score of 12) [10]. Repeat head CT demonstrated ventricular ependymal enhancement and intraventricular debris, diagnostic of ventriculitis. Repeat LP showed persistent pleocytosis, but all cultures and repeat PCR assays were negative (Table 3).

**Table 3:** CSF microbiology *Multiplex PCR testing was performed on the CSF sample collected on hospital day 1. Results became available on hospital day 3. CSF, cerebrospinal fluid; HSV-1, herpes simplex virus type 1; PCR, polymerase chain reaction; *S. pneumoniae*, *Streptococcus pneumoniae.*

Sample	Day 1	Day 14
Blood culture	*S. pneumoniae* detected	No growth
CSF Gram stain	Gram-positive cocci in chains	No organisms observed
CSF *S. pneumoniae*	Detected	Not detected
CSF HSV-1 PCR	*Detected	Not detected

Empirical meropenem was added to the regimen. The patient completed 21 days of acyclovir and a tailored course of antibiotics. He showed progressive clinical and radiological improvement and was discharged neurologically intact.

## Discussion

The interaction between *S. pneumoniae* and HSV-1 detection in the CSF represents a diagnostically complex scenario. Pneumococcal meningitis is known to cause profound blood-brain barrier disruption through an intense inflammatory cascade [11]. This disruption may create a permissive environment for the transient passage of viral DNA into the CSF or facilitate viral reactivation [5,7]. Furthermore, systemic inflammation and the associated immune dysregulation observed in severe sepsis can impair antiviral surveillance mechanisms, potentially triggering latent virus activity [12,13].

In our patient, the temporal association between early HSV-1 detection and a new-onset seizure, alongside MRI findings involving the medial temporal regions, raised concern for active viral involvement rather than isolated "bystander" DNA detection [2]. While these imaging findings can be observed in various encephalitic processes, they are highly suggestive of HSV-related CNS involvement in this clinical context [8]. Given the substantial morbidity associated with untreated HSV encephalitis, the reinitiation of acyclovir was a clinically justifiable precautionary measure [1,13].

The clinical course did not conform to the classical delayed biphasic reactivation model sometimes reported in the literature [5,7]. Instead, the early dual detection followed by a secondary deterioration at day 14 - characterized by ventriculitis and negative repeat PCR - suggests a secondary inflammatory complication rather than a viral-driven relapse [14,15]. Persistent ependymal inflammation following severe pneumococcal meningitis can contribute to such ventricular abnormalities even after microbiological clearance [11,14].

Multiplex PCR platforms, such as the BioFire® FilmArray® panel, have expanded our recognition of viral co-detection [15]. However, molecular detection must always be interpreted in context. Detection of HSV-1 DNA does not necessarily equate to active encephalitis, particularly in the presence of severe bacterial inflammation, where false-positive molecular results or incidental shedding can occur [16]. Clinical improvement following empirical treatment optimization underscores the necessity of a broad, cautious management strategy in the face of diagnostic uncertainty.

## Conclusions

Concurrent detection of *S. pneumoniae* and HSV-1 in CSF presents significant therapeutic challenges. This case underscores the importance of careful chronological interpretation of multiplex PCR results. While HSV-1 positivity should prompt clinical correlation rather than automatic attribution to encephalitis, early antiviral therapy remains a critical principle when clinical or radiologic features raise suspicion.

## References

[1] Rohani H, Arjmand R, Mozhgani SH, Shafiee A, Amini MJ, Forghani-Ramandi MM (2023). The worldwide prevalence of herpes simplex virus encephalitis and meningitis: a systematic review and meta-analysis. Turk Arch Pediatr.

[2] Bradshaw MJ, Venkatesan A (2016). Herpes simplex virus-1 encephalitis in adults: pathophysiology, diagnosis, and management. Neurotherapeutics.

[3] Zhu S, Viejo-Borbolla A (2021). Pathogenesis and virulence of herpes simplex virus. Virulence.

[4] Hasbun R (2022). Progress and challenges in bacterial meningitis: a review. JAMA.

[5] Ericsdotter AC, Brink M, Studahl M, Bengnér M (2015). Reactivation of herpes simplex type 1 in pneumococcal meningitis. J Clin Virol.

[6] Sediri S, Madika AL, Baguet JP, Mallart A, Charley-Monaca C, Mounier Vehier C (2018). [Obstructive sleep apnea syndrome in woman: knowing its specificities for a better management]. Presse Med.

[7] Martins A, Silva C, Silva F, Ribeiro L, Cruz AJ, Ceia F, Santos L (2026). Streptococcus pneumoniae and herpes simplex virus-1 central nervous system co-infection. Adv Infect Dis.

[8] Hachad R (2025). Mixed Streptococcus pneumoniae and herpes simplex virus meningoencephalitis 
confirmed by PCR: a case report and review of the literature. World J Adv Res Rev.

[9] Hamieh A, Elias R, Selim H, Elias J (2025). Bacterial or viral: a case of central nervous system infection in a young, immunocompetent adult. Cureus.

[10] Teasdale G, Jennett B (1974). Assessment of coma and impaired consciousness: a practical scale. Lancet.

[11] Salimi H, Klein RS (2019). Disruption of the blood-brain barrier during neuroinflammatory and neuroinfectious diseases. Neuroimmune Diseases: From Cells to the Living Brain.

[12] Piret J, Boivin G (2020). Immunomodulatory strategies in herpes simplex virus encephalitis. Clin Microbiol Rev.

[13] Klein RS, Garber C, Funk KE (2019). Neuroinflammation during RNA viral infections. Annu Rev Immunol.

[14] Klein M (2019). Neuroinflammation during RNA viral infections. Annu Rev Immunol.

[15] Perlejewski K, Radkowski M, Rydzanicz M (2023). Metagenomic search of viral coinfections in herpes simplex encephalitis patients. J Neurovirol.

[16] Sasan MS (2012). False positive PCR for herpes simplex virus (HSV) in childhood CNS infections. Iran Red Crescent Med J.

